# Glutathione restoration: a sword to combat skeletal muscle stem cell aging

**DOI:** 10.1093/lifemeta/load012

**Published:** 2023-03-31

**Authors:** Zeming Wu, Jie Ren, Guang-Hui Liu

**Affiliations:** State Key Laboratory of Membrane Biology, Institute of Zoology, Chinese Academy of Sciences, Beijing 100101, China; Institute for Stem Cell and Regeneration, Chinese Academy of Sciences, Beijing 100101, China; Beijing Institute for Stem Cell and Regenerative Medicine, Beijing 100101, China; Institute for Stem Cell and Regeneration, Chinese Academy of Sciences, Beijing 100101, China; CAS Key Laboratory of Genomic and Precision Medicine, Beijing Institute of Genomics, Chinese Academy of Sciences and China National Center for Bioinformation, Beijing 100101, China; University of Chinese Academy of Sciences, Beijing 100049, China; Sino-Danish College, University of Chinese Academy of Sciences, Beijing 101408, China; State Key Laboratory of Membrane Biology, Institute of Zoology, Chinese Academy of Sciences, Beijing 100101, China; Institute for Stem Cell and Regeneration, Chinese Academy of Sciences, Beijing 100101, China; Beijing Institute for Stem Cell and Regenerative Medicine, Beijing 100101, China; University of Chinese Academy of Sciences, Beijing 100049, China; Advanced Innovation Center for Human Brain Protection, and National Clinical Research Center for Geriatric Disorders, Xuanwu Hospital Capital Medical University, Beijing 100053, China; Aging Translational Medicine Center, International Center for Aging and Cancer, Xuanwu Hospital, Capital Medical University, Beijing 100053, China

**In a recent study published in *Cell Metabolism*, Thomas A. Rando and colleagues reported a critical role of dysregulated glutathione (GSH) metabolism in driving the aging process of skeletal muscle stem cells (MuSCs), uncovering a novel mechanism underlying the divergent responses of quiescent stem cells to environmental stressors with age, thus providing a potentially accessible target to alleviate age-associated skeletal muscle degeneration.**

Aging is a complex physiological process characterized by a wide range of degenerations in various tissues—this is considered a major risk to the development of many age-related diseases, including metabolic, cardiovascular, and neurodegenerative disorders [1]. However, the inherent complexity and heterogeneity of the aging process make pinpointing the causal biological alterations a daunting task, so researchers have been resorting to multiomics to understand this process, yet integrating the massive information itself is also a challenge.

Understanding the exact role of metabolic pathways in aging is a typical example of the conundrum. Metabolic dysfunction has long been considered an important hallmark and driver of aging, hence serving as a target to ameliorate aging and aging-related disorders. However, there are only a few metabolites with clear evidence for a causal relationship with aging. Among them, high levels of branched-chain amino acids (BCAAs) in plasma are associated with an increased risk of age-associated diseases in humans, while low BCAA consumption extends the life span of progeroid mice, attenuates frailty, and improves the metabolic health of wild-type C57BL/6J mice when started in midlife [2]. Similar to BCAAs, methionine restriction modifies the lipid profile and extends the healthspan and lifespan in progeroid mice [3], while inhibition of MAT2A, a methionine adenosyl-transferase catalyzing the transformation of S-adenosyl methionine from methionine, decreases ammonia levels, relieves insulin resistance, and promotes skeletal muscle regeneration [4]. These findings warrant further investigation to understand other precise ingredients of a protein-restricted diet mediating the beneficial effects on metabolic health and longevity.

Skeletal muscle stem cells (MuSCs), also known as satellite cells, are a regenerative adult stem cell population essential for muscle regeneration but decline significantly during aging. Despite numerous efforts in dissecting intrinsic and extrinsic factors that contribute to MuSC aging and developing potential interventions to combat age-associated muscle degeneration, this field is still an open question, especially from the perspective of metabolism to explain the shifts into heterogeneity in quiescent MuSCs during aging. In this issue of *Cell Metabolism*, Benjamin *et al*. addressed the knowledge gap by profiling MuSCs from young (4 months) and old (22 months) mice [5]. To characterize aging-related changes at multiple molecular levels, the authors generated a multiomic dataset covering transcriptomic, proteomic, metabolomic, and epigenomic data. Their initial analysis suggested decreased glutathione (GSH) at the metabolite level but increased oxidative stress and GSH biosynthesis at the epigenomic, transcriptomic, and proteomic levels, which appears to be an incomplete compensation signature at first glance. Further integrative analysis with a pathway rank aggregation strategy identified that GSH metabolism was at the top of the consensus list. Although it is not surprising to observe decreased GSH content with aging in rodents as revealed by previous studies [6], subsequent live-cell staining with a molecular probe targeting GSH yielded a surprising dichotomy in aged MuSCs, exhibiting normal and low GSH levels (GSH^high^ and GSH^low^) in the same population. In contrast, young MuSCs exhibited a homogeneously normal level of GSH. Moreover, the ratio of GSH^high^ to GSH^low^ MuSCs decreased significantly with age, suggesting the dynamic heterogeneity of MuSCs during aging. Subpopulation analysis in aged MuSCs showed that GSH^high^ cells possess higher proliferative potential and mitochondrial capacity than GSH^low^ cells. *In vivo* transplantation of GSH^high^ and GSH^low^ MuSCs into injured skeletal muscles of 4-month-old recipient mice confirmed that cells with higher levels of GSH are more effective in myogenesis. These results lay the groundwork for promoting MuSC self-renewal and muscle regeneration by supplementation with GSH.

Considering that cellular GSH content is largely determined by *de novo* synthesis and regeneration from oxidative GSH (glutathione disulfide, GSSG), the authors manipulated the GSH content by supplying N-acetylcysteine (NAC), a cysteine precursor, to promote the *de novo* synthesis of GSH or with 6-aminonicotinamide, a glucose-6-phosphate dehydrogenase inhibitor, to prevent NADPH-mediated reduction of GSSG to GSH [7, 8]. As a result, increasing GSH content promoted the survival and proliferation potential of MuSCs *in vitro*. More importantly, NAC supplementation by intramuscular injection or in drinking water improved the regenerative capacity of skeletal muscles in aged mice, paving the way for small molecule-based strategies to combat skeletal muscle aging *in vivo*.

Finally, to uncover the molecular mechanism underlying the bimodal signature of GSH content in aged MuSCs, the transcriptomic differences in young MuSCs, GSH^high^ aged MuSCs, and GSH^low^ aged MuSCs were explored to identify key regulators in this process. Intriguingly, the authors observed a higher enrichment of genes related to GSH biosynthesis in GSH^high^ cells than in the GSH^low^ population, and thus proposed a new view that the compensatory signature observed in the multiomics analysis at the bulk level is driven by a subpopulation of the GSH^high^ MuSCs. Further analysis identified that nuclear factor erythroid 2-related factor 2 (NRF2), the well-known antioxidant transcription factor involved in GSH metabolism [9], was highly enriched in GSH^high^ cells. In contrast, enrichment of nuclear factor kappa-B (NF-κB), a well-known transcription factor involved in promoting inflammation and aging [10], was found in GSH^low^ MuSCs. NF-κB activation could repress NRF2 activation and GSH synthesis, while inhibition of NF-κB increased NRF2 target gene expression, GSH content, and the ratio of GSH^high^ to GSH^low^ MuSCs. Accordingly, these findings suggest that the mutual antagonism between NRF2 and NF-κB mediates the bimodal signature of MuSC aging.

**Figure 1:**
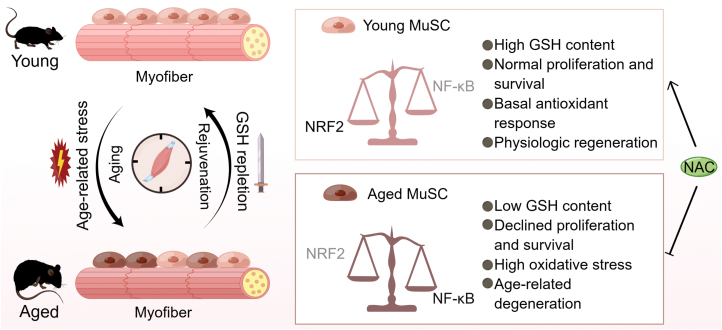
Schematic diagram showing the bimodal signature and underlying mechanism of skeletal MuSC aging. During skeletal muscle aging, age-related stresses induce the progressive decline of skeletal muscle, leading to heterogeneity of aged MuSCs, decline of regenerative capacity, and atrophy. Meanwhile, this process can be reversed by the repletion of GSH, which acts as a sword to combat skeletal muscle aging. In detail, the dichotomic population (heterogeneity) of aged MuSCs is characterized by subpopulations harboring normal and low GSH levels (GSH^high^ and GSH^low^), while young MuSCs present a homogeneously normal level of GSH. In the younger state, NRF2 activation contributes to high GSH content, normal cellular proliferation and survival, basal antioxidant response, and physiologic regeneration. However, MuSCs in the aged state endure NF-κB activation and exhibit low GSH levels, impaired proliferative capacity and cell survival, high oxidative stress, and progressive degeneration. The mutual antagonism between NRF2 and NF-κB mediates the bimodal signature of aged MuSCs, which can be rejuvenated by restoring GSH synthesis via supplementation with NAC. The schematic diagram is prepared by Figdraw.

In summary, Benjamin *et al*. revealed a stressor-response model of MuSC aging, providing phenotypic evidence and mechanistic insights into the heterogeneity of stem cell aging (Fig. 1). In this model, young MuSCs are exposed to relatively low levels of oxidative stress and therefore show minimal signatures of the oxidative stress response. These cells are rich in GSH and function well. Conversely, aged MuSCs are exposed to oxidative stress and respond in a bimodal signature. On the one hand, GSH^high^ MuSCs generate a compensatory response by upregulating NRF2 activity and GSH synthesis. These cells are enriched in GSH and retain functionality in the face of aging-related stresses, especially the oxidative stress. On the other hand, in GSH^low^ MuSCs, NRF2 activity is repressed by NF-κB, which inhibits GSH synthesis and leads to regenerative dysfunction. Through manipulation of GSH metabolism, this study sheds new light on the development of potential intervention approaches (e.g., NAC supplementation) to improve MuSC homeostasis and to combat skeletal muscle degeneration. In the future, from a systemic point of view, it will be very interesting to see if the same metabolite can target the heterogeneous functionality of other adult stem cells, which are the foundation of tissue homeostasis and regeneration across the body, to achieve a systemic effect on the whole organism.
